# Error Separation Method for Geometric Distribution Error Modeling of Precision Machining Surfaces Based on K-Space Spectrum

**DOI:** 10.3390/s24248067

**Published:** 2024-12-18

**Authors:** Zhichao Sheng, Jian Xiong, Zhijing Zhang, Taiyu Su, Min Zhang, Qimuge Saren, Xiao Chen

**Affiliations:** 1 School of Mechanical Engineering, Beijing Institute of Technology, Beijing 100081, China; zxy71677@163.com (Z.S.);; 2 EPSRC Future Metrology Hub, University of Huddersfield, Huddersfield HD1 3DH, UK; 3 Genertec Machine Tool Engineering Research Institute Co., Ltd., Beijing 101300, China

**Keywords:** error separation, geometric distribution error, skin model, K-space spectrum, random error filtering

## Abstract

The geometric error distributed on components’ contact surfaces is a critical factor affecting assembly accuracy and precision instrument stability. Effective error separation methods can improve model accuracy, thereby aiding in performance prediction and process optimization. Here, an error separation method for geometric distribution error modeling for precision machining surfaces based on the K-space spectrum is proposed. To determine the boundary of systematical error and random error, we used a cruciform boundary line method based on the K-space spectrum, achieving the optimal separation of the two with frequency difference. The effectiveness of the method was experimentally verified using two sets of machined surfaces. By comparing with current common random error filtering methods, the outstanding role of the proposed error separation method in separating random error and preserving processing features has been verified.

## 1. Introduction

In precision instruments, geometric distribution error (GDE) [1] affects the accuracy and stability of assembled products. The analysis and processing method of GDE data, especially on how to effectively separate systematical error and random error, is an urgent problem in establishing a precision electromechanical product’s assembly contact surface GDE model and optimizing the assembly process [2].

For example, Qimuge’s precise geometric digital twin modeling method [3] adopts the GDE modeling approach. Based on measured data, Qimuge retains machining feature data after error separation and constructs a three-dimensional solid model through surface interpolation. Finally, by using the finite element method for virtual assembly, the prediction and control of assembly accuracy and stability are achieved [4,5]. This type of method can be used for precision machine tools, gears, and other components, optical systems [6] and other precision equipment’s accuracy and performance prediction and control. The measurement, separation, and processing of errors constitute its direct data foundation.

Regarding the analysis of GDE in the measurement data of machining surface [7], its sources can be divided into three parts. One is the error caused by the measurement process, which needs to be adjusted in order to reduce the error as much as possible in preprocessing. Another type is machining features generated during machining, which are prominent features brought by specific machining methods, such as dents in grinding and tool marks in milling. This error is a systematical error, which is inevitable and regular in machining. It is a key influencing factor on the assembly accuracy of precision instruments and is also the datum directly used in modeling. Finally, there is the random error, mainly high-frequency random error, including slight tool vibration, burrs, and debris, as well as random disturbances in measurement results.

We expect to remove other errors while preserving processing features as much as possible; different types of GDE need to be processed. On the one hand, we need to handle the error caused by the measurement process. This aspect belongs to the preprocessing and is not the focus of this article’s research. On the other hand, we need to separate random error, and the separation of random error in machining surfaces has significant specificity.

High-precision methods are required in measurement. The single point measurement method’s accuracy is significantly higher than scanning. However, due to high measurement costs, we can only obtain sparse data through single-point measurement, with a spacing of approximately 1 mm or 0.1 mm. Although there are currently data recovery methods based on sparse data, such as Sun’s network-based 2.5D point cloud recovery method [8], these methods heavily rely on datasets and have a significant gap in real-world use. Moreover, in GDE, it is difficult to find clear feature boundaries. And the error data are more microscopic compared to normal point clouds. The magnitude of random error can reach a level similar to machining features.

Based on the particularity of separating random error in machining surfaces, this paper analyzes the main methods and recent studies for separating and removing random error and their application effects in a GDE processing scenario.

One way is through the surface fitting methods, which are mainly used in point cloud filtering and reverse engineering [9]. Using surface fitting methods that do not completely pass through all control points can result in filtering random error. The specific fitting methods are divided into multiple categories based on the different fitting formulas; a classic method is polynomial fitting, which includes the use of local quadratic surface fitting to improve fitting accuracy [10]. However, this method is mainly suitable for smooth surfaces; machining surfaces often have sharp features such as tool marks and grooves, which are difficult to preserve under this method. Current research is mainly related to free curve fitting, such as the combined method of Lagrange operator and least square method [11], or the combination of B-spline fitting and least square method [12,13], which inherits the advantages of geometric iteration method (PIA) while achieving B-spline fitting. However, these methods are essentially an approximation and smoothing approach. When data points with significant errors are handled, they are usually removed based on algorithms such as RANSAC [14]. For situations where measurement data are sparse, and when the difference in magnitude between systematical error and random error is not significant, it is difficult to achieve substantial differentiation between the two types of error.

Another way of removing random error is through statistical methods, which directly achieve smoothing effects in linear or nonlinear forms based on data values and are widely used in image filtering or point cloud filtering. Gaussian filtering and median filtering belong in this category, but the disadvantage is that they can easily lead to the elimination of both features and random error. Multiple methods have emerged to address this issue, including the nonlocal mean method, which utilizes image redundancy [15] and can be combined with the idea of variable scaling [16]. Alternatively, bilateral filtering methods that protect boundaries from excessive smoothing can be combined with adaptive methods [17] or with the idea of variable scaling [18]. However, whether for nonlocal mean methods or adaptive methods, it is necessary to identify obvious features, which are not realistic at low data densities.

Frequency based methods are also widely used methods for separating systematical error and random error, which are mainly low-pass filtering and wavelet filtering methods. The implementation of the low-pass filtering method is simple, but the effect is usually poor due to the loss of all high-frequency signals. However, wavelet filtering has the problem of uncertain wavelet bases, and different wavelet bases will bring different filtering effects [19]. Although some scholars have applied wavelet filtering to actual machining surfaces [20], its specific application scenarios involve dense scanning point cloud data with low accuracy requirements. When the data are sparse, it is difficult to finely decide frequency boundaries.

Various deep learning methods have also been applied in this regard [21]. The main approach is the combination of other filtering methods with neural networks, such as the combination of wavelet transform with neural networks [22] or the combination of surface fitting with neural networks [23]. However, due to the high cost of processing precision instruments, it is difficult to obtain a sufficiently large dataset for the application scenario for processing GDE.

In summary, existing filtering methods for surface and point clouds are often unable to meet the high-precision separation requirements of GDE. Therefore, an error separation method for the geometric distribution error modeling of precision machining surfaces based on the K-space spectrum (K-GDES) is proposed, which is frequency-based and can determine the frequency boundary between random error and systematical error in the machining surface error with evidence. Firstly, the overall framework of the method is proposed, based on which the K-GDES is divided into three parts, including preprocessing, frequency domain representation, identification, and separation. Under the premise of preprocessing, a K-space spectrum representation method for machining surface error is proposed to achieve frequency domain data transformation. On this basis, we set a cruciform boundary line for systematical error and random error by analyzing the mapping relationship between error and the K-space spectrum. In response to the difficulty in clearly defining the frequency limit of systematical error and random error, an identification method based on error distribution similarity calculation is proposed. After the boundary line is determined, systematical error and random error can be effectively separated. In addition, the method proposed is validated by using the data of the machining surface, which was machined in the same machining method. The results show that under low sampling data density, the separation effect of this method is superior to the common methods in terms of accuracy and processing feature retention ability. This method is particularly adept at preserving periodic processing feature information and is particularly sensitive to stripe-like features.

K-GDES requires obtaining batch machining error measurement data, which refers to the measurement data of multiple machining surfaces obtained by the same machining method. This method can be used for identifying and extracting the regularity features and randomness features of batch machining surface data, which achieves the separation of systematical error and random error and uses the separation results to construct skin models. This study provides an error separation method for handling geometric distribution errors and a geometric modeling method for improving the effectiveness of the model.

## 2. The Framework of K-GDES Method

**Figure 1:**
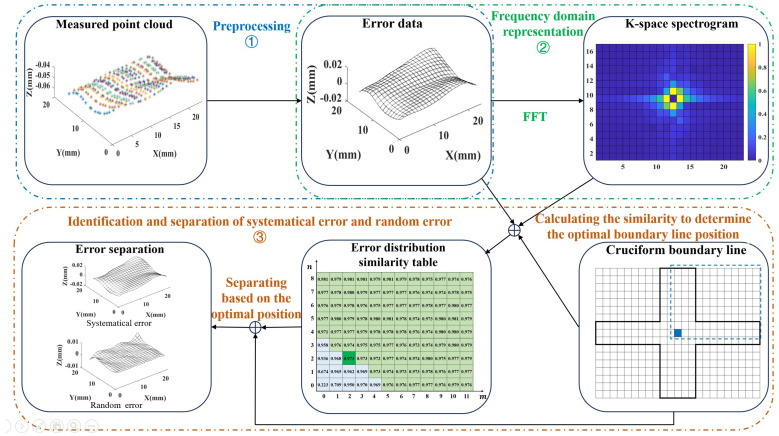
K-GDES method framework diagram.

K-GDES is divided into three parts: preprocessing, frequency domain representation, identification, and separation. By transforming error data into K-space spectrum information, it achieves effective recognition and separation of systematical error and random error. Preprocessing to extract GDE from a series of machining surfaces and perform uniform interpolation to connect with subsequent methods. Frequency domain representation uses two-dimensional Fast Fourier Transformation (FFT) to transform the error data into a K-space spectrum. The cruciform boundary line method is proposed for error identification and separation, and the optimal cruciform boundary position is determined by calculating the error distribution similarity. The brief framework is shown in Figure 1:

Analyze Figure 1. Firstly, preprocess the measurement data, as shown in ➀, to transform the machining surface measurement data into uniformly interpolated error data. The essence of error data is still in the form of point clouds, but it presents a uniform mesh surface shape, so it can also be called an error surface. Preprocessing includes separating and removing errors caused by measurement, obtaining error data by subtracting the ideal surface from the machining surface, and uniformly interpolating the error point cloud. Then, as shown in ➁, the frequency domain representation of the error data is performed, and the K-space spectrum is obtained using FFT. Finally, as shown in ➂, the identification and separation are carried out to achieve optimal separation of systematical error and random error based on frequency. This section ➂ includes the cruciform boundary line method, the determination of the optimal boundary position based on calculating the error distribution similarity, and the final separation of systematical error and random error.

Summarize the process of K-GDES. Firstly, preprocess the original point cloud measurement data of a batch of machining surfaces machined by the same machining method to obtain error data. Then, through FFT, the K-space spectrums are obtained. By analyzing the mapping relationship between two kinds of errors and the K-space spectrum, a separation method based on the cruciform boundary line is determined, and the error distribution similarity calculation method is proposed to obtain the optimal position of the boundary line. Finally, based on the obtained optimal boundary position, the separation of a batch of error data is achieved. The foundation of K-GDES lies in the frequency domain of machining error, and the core lies in quantitatively characterizing the similarity of GDE in the same batch of machining surfaces and exploring the numerical influence of systematical error and random error on this similarity.

## 3. Preprocessing and Frequency Domain Representation

Preprocessing realizes the transformation of the original point cloud measurement data of the machining surface to the error point cloud of uniform interpolation. After obtaining the uniform error point cloud data, it is characterized in the frequency domain and subjected to systematical error and random error separation processing in the frequency domain. In K-GDES, the distinction between systematical error and random error is mainly based on their frequency differences, so the frequency-domain method can used for separation. Systematical error usually brings about large-scale morphological changes, often containing more low-frequency components, while random errors are usually slight in form but numerous in quantity, tending toward high frequencies.

### 3.1. Preprocessing

Preprocessing includes separating and compensating for errors caused by measurement, obtaining error data by subtracting the ideal surface from the machining surface, and constructing uniformly distributed error point cloud data.

#### 3.1.1. Compensating for the Error Caused by Measurement

The error caused by measurement is not the error we need when analyzing GDE and modeling, and the error caused by measurement cannot be distinguished from machining features by frequency. So it is necessary to reduce them before separating random error. The error caused by measurement can be divided into method error, instrument error, environmental error, and human error.

Method error mainly involves the selection of measurement benchmarks and the sampling method. How to choose a suitable measurement method is usually determined by drawing requirements, assembly requirements, and measurement experience. In the application scenario of this article, the measured surface can be set as the measurement benchmark, and uniform grids can be taken as measurement points to standardize the measurement process.

Instrument error refers to the error of the measuring machine itself, which can be divided into two parts. The first part comes from the error caused by the accuracy problem of the measuring equipment, which can be obtained by measuring a combination of three axial and four spatial directions of measuring blocks. This error gradually increases with the use of the machine, and maintenance and regular calibration of the equipment can effectively reduce the variation of this error [24]. The second part is the error caused by the probe and needle components [25], including the rotational positioning error of the rotating measuring seat and the error caused by various adapters and joints. To reduce this part of the error, it is necessary to calibrate the measuring needle and choose the shortest possible probe and needle group. Using laser-based rather than contact-based measuring machines can also effectively avoid the error caused by the probe and needle components.

Environmental error refers to the variation in measurement values caused by the different environments in which the workpiece and measuring machine are located, with temperature and vibration being the most significant influencing factors. There are various methods to reduce the error caused by temperature changes: first, ensuring a constant temperature in the measurement environment; second, controlling the temperature of the measured workpiece; third, using the temperature compensation function of the measuring machine for automatic compensation. Vibration also has a significant impact on measurement accuracy, measuring machines cannot be installed in areas with strong seismic sources and high noise.

Human error mainly occurs during manual measurement. The difference in measurement force during manual measurement leads to human error. Another reason is that it is difficult to manually measure points according to the vector direction during sampling, resulting in human error. Therefore, it is necessary to use automated measuring machines to avoid this error.

In summary, in order to reduce the error caused by measurement, various measures need to be taken. On the one hand, it is necessary to calibrate the measuring machine, including the accuracy calibration of the measuring machine itself, the calibration of the probe and needle components, and the calibration of the measuring platform. On the other hand, it is necessary to ensure the stability of the measurement environment and reduce the influence of temperature and vibration factors. Finally, data compensation should be performed as needed. The compensation method can be either the built-in compensation of the measuring machine or additional compensation during data processing, including temperature compensation and compensation for measurement platform offset. When measuring a rotating body, it is also necessary to consider the offset between the central axis of the rotating body and the central axis of the turntable, as well as the compensation for error in the turntable itself.

#### 3.1.2. Obtaining Error Data

After removing the error caused by the measurement process, it is necessary to use ideal surface data for subtraction to translate surface measurement data to error data. When the ideal surface is a plane, the undulating features directly measured on the machining surface are exactly the same as the error undulating features. But when the surface is a curved surface, the measurement data of the machining surface needs to substract the ideal surface in order to obtain the fluctuation characteristics of the error surface. When the machining surface is a rotating surface such as a threaded surface, in addition to substracting the ideal surface data, it should also be unfolded along the axis to more intuitively display the error characteristics. In this article, to visually demonstrate K-GDES and its effectiveness, only to use the plane ideal machining surface as example.

#### 3.1.3. Constructing Uniformly Distributed Error Data

**Figure 2:**
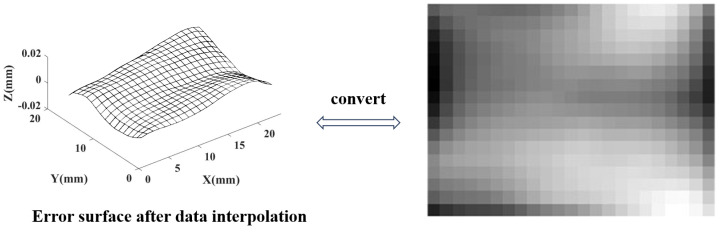
Uniformly distributed error data and their equivalent grayscale image.

Discrete frequency domain transformation requires an equidistant distribution of data points, but the obtained error point cloud data may not be uniformly distributed due to the diversity of measurement schemes. In order to connect with FFT, uniform interpolation is applied to the error data. In the direction of X and Y, with a self set spacing of $d_{x}$ and $d_{y}$, a uniform interpolation on the error data is performed. This error surface can be mapped to a two-dimensional grayscale image, thereby connecting with the FFT method. As shown in Figure 2, $d_{x}=d_{y}$ = 1 mm.

### 3.2. K-Space Spectrum of GDE

**Figure 3:**
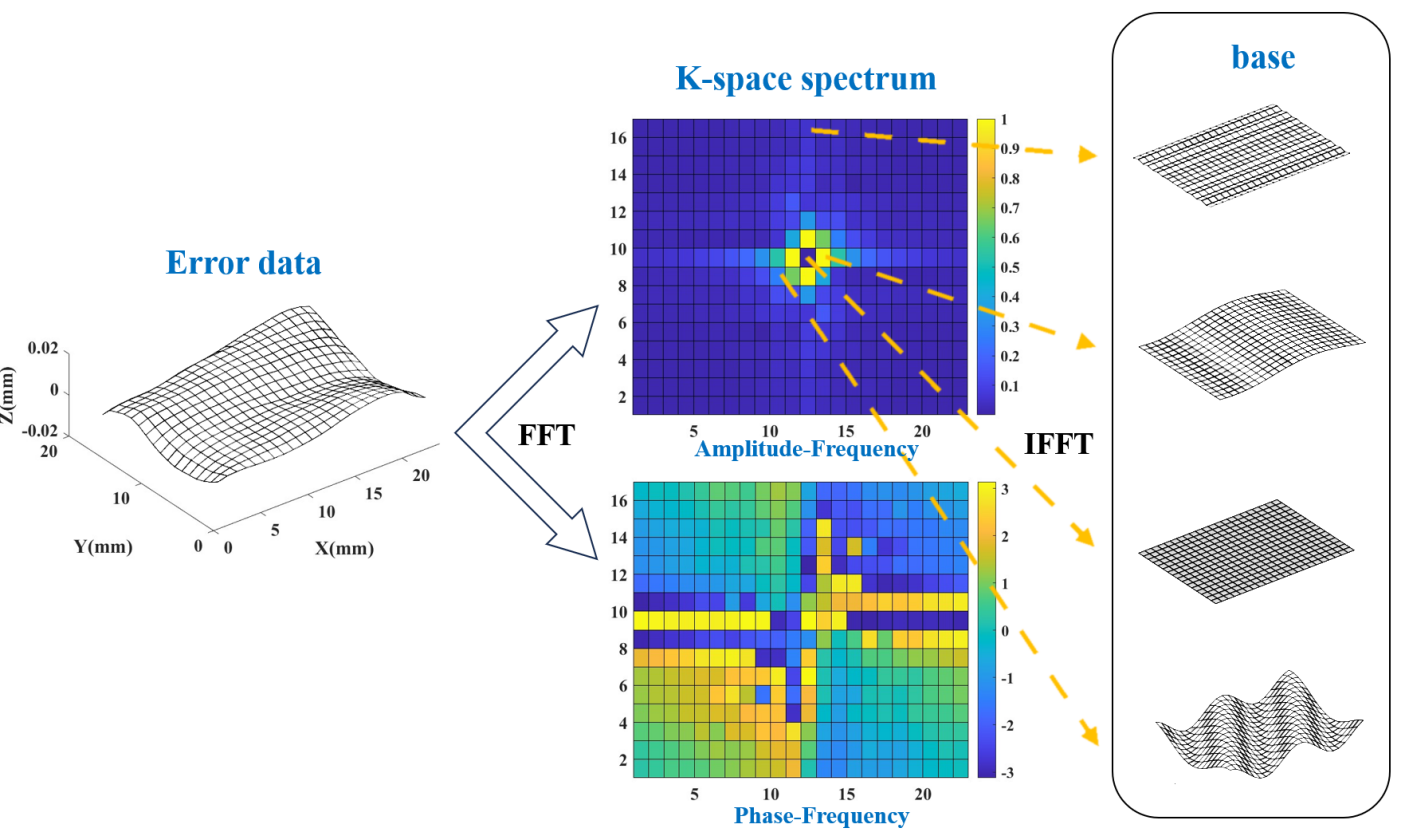
K-space spectrum, elements, and bases.

The formula for FFT and inverse transform IFFT refers to [26]. K-space refers to the frequency space of the Fourier transform, the K-space spectrum is the result of FFT and also the specific manifestation of error data at the frequency domain level. The size of the K-space spectrum is as same as the number of discrete points in the error data after uniform interpolation, set as M × N. The K-space spectrum includes the K-space amplitude spectrum (amplitude–frequency spectrum) and the K-space phase spectrum (phase–frequency spectrum). Each block on the K-space spectrum is called an element, and each element has its physical meaning. The K-space amplitude spectrum is symmetric about the center, and the value of each element represents the amplitude, while the position of the element represents the frequency. The K-space phase spectrum is about center negative symmetry, and the value of each element represents phase, the position of element also represents frequency, and correspond to the K-space amplitude spectrum. Each element corresponds to a combination of amplitude–phase–frequency, which can be used as a base to form the original error surface after inverse transformation. The error surface is a superposition of M×N bases. The K-space spectrum obtained by FFT is shown in Figure 3.

Analyze the X and Y directional frequencies represented by the element position in the K-space spectrum. When the data size is M × N, with data spacing of $d_{x}$ and $d_{y}$ respectively, any element has a distance (a,b) from the center element. The frequency represented by the position of this element in the X and Y directions as Equations (1) and (2):

(1) $$P_{X}\left(a,b\right)=\frac{a}{Md_{x}}$$

(2) $$P_{Y}\left(a,b\right)=\frac{b}{Nd_{y}}$$

where, $a=0,1,\ldots ,\left\lfloor M/2\right\rfloor$, $b=0,1,\ldots ,\left\lfloor N/2\right\rfloor$.

The separation of systematical error and random error mainly needs the K-space amplitude spectrum. The phase is mainly affected by the feature position and has high randomness after FFT. Therefore, only the K-space amplitude spectrum will be shown in the subsequent figures. When processing the spectrum, it is also based on the K-space amplitude spectrum. But when performing IFFT, it is necessary to use the unmodified K-space phase spectrum.

## 4. Identification and Separation

To achieve optimal separation of systematical error and random error based on frequency, the distribution characteristics of the two types of errors on the K-space spectrum are first discussed. Through the mapping relationship between the K-space spectrum and the two types of errors, the cruciform boundary line method is proposed. Subsequently, the optimal position of the cruciform boundary line is determined by calculating the error distribution similarity, and based on this position, systematical error and random error are separated.

### 4.1. Cruciform Boundary Line

As discussed in Section 3.2, the error surface is the superposition of bases, with some bases overlapping to form systematical error and another part of bases overlapping to form random error. It is necessary to identify which elements are inverted to form systematical error and which elements form random error.

**Figure 4:**
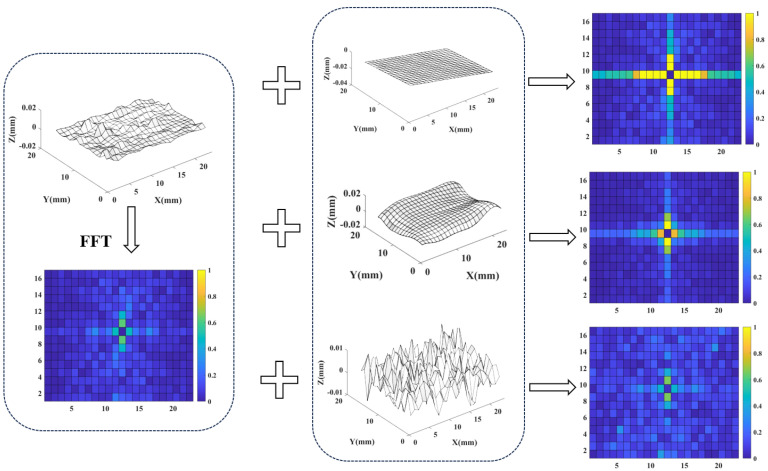
Diagram of the correspondence between the K-space spectrum and error surface.

By analyzing the example shown in Figure 4, explore the relationship between two kinds of errors and the K-space spectrum. When the error surface superimposes a tilted plane, the K-space amplitude spectrum only undergoes significant changes in one row and one column, which crosses the center element. When the error surface superimposes a smooth surface with significant shape changes, the K-space amplitude spectrum only undergoes significant changes in several rows and columns near the center. When the error surface superimposes a highly fluctuating random surface, the K-space amplitude spectrum changes significantly near the four corners.

It can be seen that the boundary between systematical error and random error in the spectrum could be a cruciform boundary line. As is shown in Equations (1) and (2), any element in the K-space spectrum, the closer it is to the central element in the X or Y direction, the lower the frequency in that direction. Therefore, low-frequency elements in the X and Y directions will jointly present a cross shape containing the central row and sequence. The base superposition of the elements contained in this cross shape will form the systematical error, while the base superposition of the elements near the corners will form the random error.

The cruciform boundary line method is essentially a frequency based approach, which determines the beneficial effects and limitations of K-GDES. On the K-space spectrum, using a cruciform boundary line to distinguish between systematical error and random error essentially divides only the part which both has a high frequency in the X and Y directions into random error. This part includes large errors that occur at individual measurement points, small protrusions (usually debris) that appear separately, and high-frequency noise. What can be preserved are the surface shape information with two directions being low-frequency and the machining features with at least one direction being low-frequency, including tilting, tool marks, and stripes. Therefore, K-GDES has good preservation ability for strip features and low-frequency features in the machining surface but cannot handle low-frequency random error.

### 4.2. Optimal Cruciform Boundary Position Determine Method

The method for determining the optimal position of the cruciform boundary line is based on the representation method of the cruciform boundary position and the calculation method of error distribution similarity. The method’s logic is as follows. In theory, a systematical error has good repeatability and large amplitude, which has a significant impact on the degree of feature similarity. The randomness of random errors is relatively high, while their amplitude is low, and their impact on the feature similarity should exhibit a slight and random characteristic. Therefore, for a batch of error surfaces, reducing the random error of one error surface will result in fluctuations in the similarity between this surface and the other error surfaces rather than significant changes. The method for determining the optimal position of the cruciform boundary line requires continuous adjustment of the position of the cruciform boundary line and calculation of the error distribution similarity. Form an error distribution similarity table and identify the boundary position between slight and large fluctuations in similarity in this table. Use this position as the optimal frequency boundary for systematical error and random error.

#### 4.2.1. Representation Method of Cruciform Boundary Position

**Figure 5:**
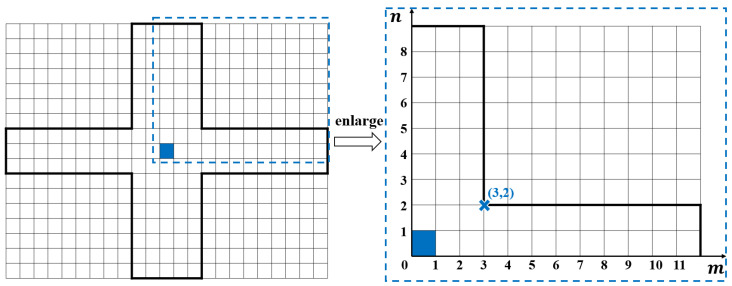
The coordinate representation method of the cruciform boundary line.

A representation method is proposed to represent the position of the cruciform boundary line. Due to the fact that the boundary line on the K-space amplitude spectrum should be symmetrical in the X and Y directions, a quarter area of the spectrum can be numbered. Let (m,n) be the coordinates of the turning point on the upper right side of the cruciform boundary line, as shown in Figure 5.

When $m=1,2,\ldots ,\left\lfloor M/2\right\rfloor$ and $n=1,2,\ldots ,\left\lfloor N/2\right\rfloor$, (m,n) represents a cruciform boundary line containing 2m−1 columns and 2n−1 rows near the K-space spectrum center. When there is a coordinate value of 0, it is a special case, (0,0) represents the boundary line containing only the central element. When m=0 and $n=1,2,\ldots ,\left\lfloor N/2\right\rfloor$, (m,n) represents a rectangular boundary line containing 2n−1 rows. When n=0 and $m=1,2,\ldots ,\left\lfloor M/2\right\rfloor$, (m,n) represents a rectangular boundary line containing 2m−1 columns.

#### 4.2.2. Calculation Method of Error Distribution Similarity

There are many methods for calculating the degree of similarity or deviation between two surfaces, such as the several methods mentioned in [27]. However, in the scenario of GDE processing, this similarity should not be too sensitive to the position of machining features, and the simplicity of the method should be considered. Therefore, the following similarity calculation method is adopted. Define the error distribution similarity η to characterize the degree of feature similarity between two error surfaces. Suppose there are M × N points in each of the two error surface, denoted as $\left(x_{1i},y_{1i},z_{1i}\right)$ and $\left(x_{2i},y_{2i},z_{2i}\right)$, i=1,2,…,M×N. Divide (M×N)/5 intervals evenly, represented as $R_{j}$, as shown in Equation (3):

(3) $$\begin{array}{cc} R_{j}\in & \left[\min\left(z_{1i},z_{2i}\right)+\frac{5(j-1)}{MN}\left(\max\left(z_{1i},z_{2i}\right)-\min\left(z_{1i},z_{2i}\right)\right),\right.\\ \; & \left.\min\left(z_{1i},z_{2i}\right)+\frac{5j}{MN}\left(\max\left(z_{1i},z_{2i}\right)-\min\left(z_{1i},z_{2i}\right)\right)\right],\\ \; & j=1,2,\ldots ,\frac{MN}{5} \end{array}$$

Set the discrete probability distribution of $z_{1i}$ on interval $R_{j}$ as p(j), and set the discrete probability distribution of $z_{2i}$ on interval $R_{j}$ as q(j). Calculate the error distribution similarity using the following formula:

(4) $$\eta =\sum_{j=1}^{\frac{MN}{5}}\sqrt{p(j)*q(j)}$$

The range of η values is [0,1], and the larger the value, the more similar the features between the two error surfaces.

This calculation is essentially a variant method of calculating the similarity between two images based on the distribution of grayscale histograms. The advantage is that this method can directly calculate the similarity of the error surface using only the Z coordinate; therefore, it is not too sensitive to the position of features. And this algorithm can avoid the impact of large local deviations on the results and mainly reflect the similarity of shape trends.

#### 4.2.3. Error Distribution Similarity Table

**Figure 6:**
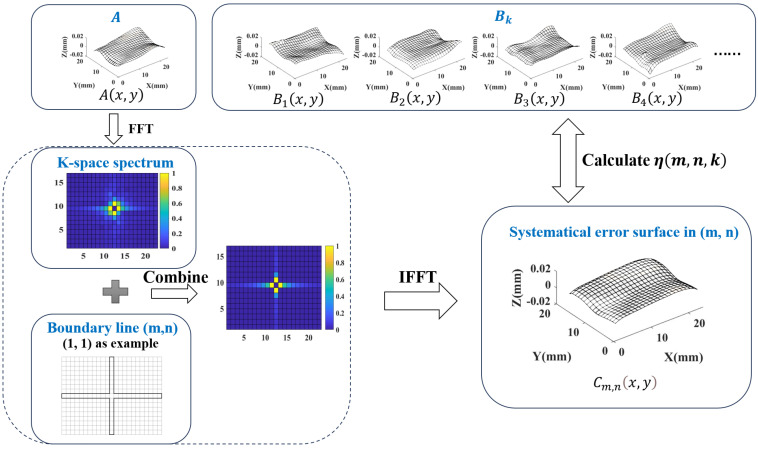
Schematic diagram for calculating $\eta \left(m,n,k\right)$.

The error distribution similarity table essentially calculates the degree of similarity between the systematical error of an error surface in different boundary positions and other error surfaces. Set there are *Q* error surfaces, take one of them as the selected surface $A\left(x,y\right)$. The other Q−1 surfaces are used as comparison surfaces $B\left(x,y\right)$, k=1,2,…,Q−1. As shown in Figure 5, the position of the boundary line is represented as (m,n). Transform m,n,k, and calculate the error distribution similarity $\eta \left(m,n,k\right)$ using the method described in Section 4.2.2. The method is shown in Figure 6.

Separate the K-space amplitude spectrum of the selected surface $A\left(x,y\right)$ using position (m,n). The internal elements of the boundary line correspond to the systematical error part, and set the external elements of the boundary line to zero. Combined with the K-space phase spectrum, use IFFT to obtain the systematical error $C_{m,n}\left(x,y\right)$ at the position (m,n). Calculate error distribution similarity between $C_{m,n}\left(x,y\right)$ and comparison surfaces $B\left(x,y\right)$. Change the position of the boundary line in sequence and calculate the error distribution similarity $\eta \left(m,n,k\right)$ between the systematical error surface and the K-1 comparison surfaces $B_{k}\left(x,y\right)$. When calculating $\eta \left(m,n,k\right)$, $C_{m,n}\left(x,y\right)$ is used as $\left(x_{1i},y_{1i},z_{1i}\right)$ in Section 4.2.2, and $B_{k}\left(x,y\right)$ is used as $\left(x_{2i},y_{2i},z_{2i}\right)$.

Take the average error distribution similarity obtained from Q − 1 comparison surfaces at the same boundary position, the similarity table H(m,n) can be obtained. The formula is Equation (5):

(5) $$H\left(m,n\right)=\frac{\sum_{k=1}^{Q-1}\eta (m,n,k)}{Q-1}$$

Due to the value of *m* or *n* can be 0, Table H(m,n) starts with H(0,0).

The selection of selected surface *A* has a relatively slight impact on the separation results, and this article analyzes the impact of the selection of *A* in Section 5.3.

#### 4.2.4. Determine the Optimal Boundary Position

**Figure 7:**
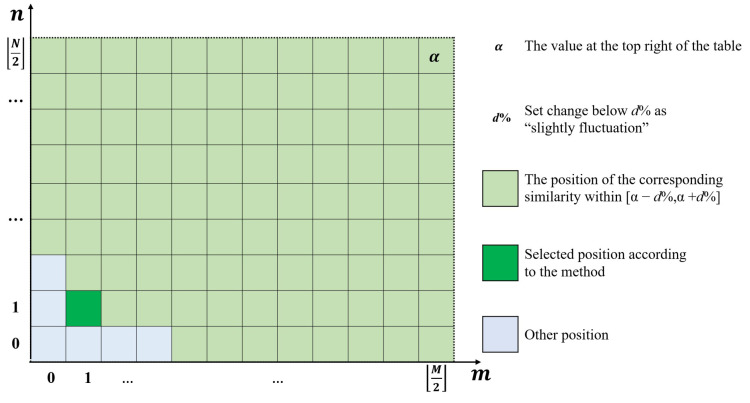
Example of error distribution similarity table.

The optimal boundary position is determined through the error distribution similarity table. As *m* or *n* increases, the values in the table H(m,n) will change from rapidly rising to slightly fluctuating. As *m* or *n* increases, the interior of the boundary line contains more systematical error, leading to an increase in error distribution similarity. The random error contained within the boundary line causes slight fluctuations of error distribution similarity. Therefore, the boundary position actual needs to be selected should be at the junction of the rapid increase and slight fluctuations. The specific selection method is shown in Figure 7:

For the specific selection rule of the optimal boundary position, the following method can be used. When m and n are maximum, the systematical error surface obtained $C_{m,n}\left(x,y\right)$ is closest to the selected surface $A\left(x,y\right)$. Therefore, the value at the top right of the table α approximates the error distribution similarity between the selected surface and the comparison surfaces. If set below d% as a slight fluctuation, mark the values in the table within [α−d%,α+d%], as shown in all green parts in Figure 7. Each marked position corresponds to a boundary position; select the marked position closest to (0,0), and if there are double-marked positions close to (0,0), choose the position near the line m/n=M/N. As shown in the figure above, the selected position is represented by a dark green color, corresponding to the boundary position of (1,1).

The final location of the boundary line is affected by the value of the slight fluctuation threshold. After multiple experiments, it is found that d% can usually be taken as 0.5% or 1%. The uncertainty of the threshold essentially stems from the ambiguity of the frequency boundary between systematical error and random error, which cannot be avoided in frequency-based methods. However, due to the junction between rapid increase and slight fluctuation can be identified effectively, the final boundary positions obtained are similar.

### 4.3. Results of K-GDES and Modeling

**Figure 8:**
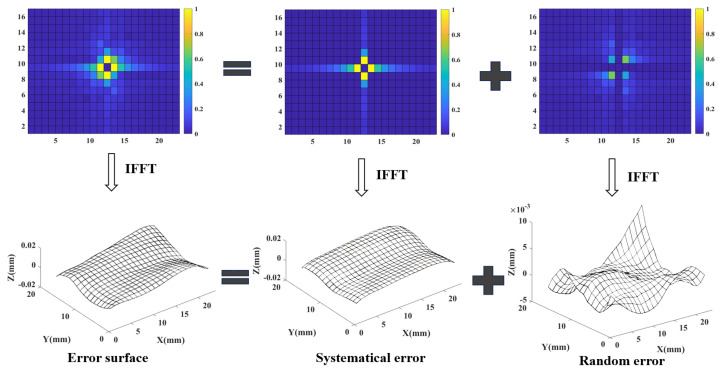
The separation results of systematical error and random error.

**Figure 9:**
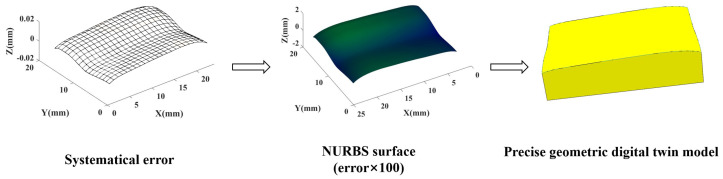
Constructing the model using separation results.

For a batch of error surfaces, after determining the boundary position, the elements outside the boundary line in their K-space amplitude spectrum are set to zero, and combined with their respective K-space phase spectrum, a batch of systematical error surfaces is obtained through FFT. On the contrary, if the elements within the boundary line in their K-space amplitude spectrum are set to zero, and the random error surfaces are obtained after FFT. Taking the hypothetical boundary position (1,1) as an example, the separation of an error surface is shown in Figure 8:

The systematical error surfaces obtained are suitable for constructing accurate geometric digital twin models considering GDE for batch machining surfaces. For specific modeling methods, as referred in [2,3], the systematical error surface is interpolated using the NURBS method and combined with the ideal model to obtain the model as shown in Figure 9.

## 5. Experimental Verification and Method Analysis

Based on measured data of machining surfaces, the error separation effect of K-GDES is demonstrated. Subsequently, the stability of the method is analyzed, mainly exploring the impact of the selection of separation surface *A*.

### 5.1. Experimental Condition

**Figure 10:**
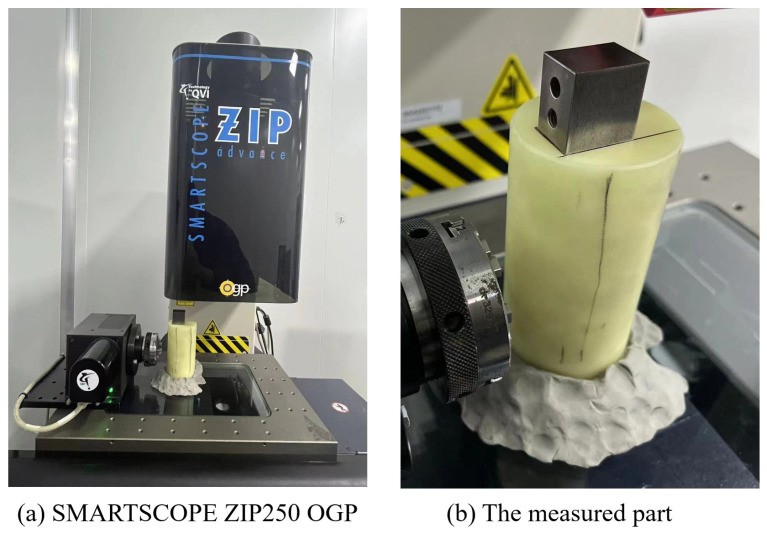
OGP measuring instrument and the measured part (using a grinding surface).

A batch of grinding surfaces and a batch of milling surfaces are measured as data sources for the experiment, with 9 surfaces in each batch. The material of grinding parts is steel 4J32. The nominal roughness of this batch of grinding surfaces is 0.8 μm. The material of milling parts is steel 45#. The nominal roughness of this batch of grinding surfaces is 3.2 μm. Each surface in one batch is machined using the same machine tool and under the same machining parameters and environmental parameters. The size of the machined surface is 25 × 18 mm, with the grinding surface polished to 12,000 mesh and the milling surface machined using CNC850. The SMARTSCOPE ZIP250 OGP measurement instrument (OGP) is used to measure the machining surface based on the laser single-point measurement method, with 23 × 17 points measured at 1mm intervals on each surface. The measurement range under OGP is 250 × 150 × 200 mm, with a measurement accuracy of 1.2 + L/250 μm in the X and Y directions and 1.9 + L/200 μm in the Z direction. L is the length of the measured part. The OGP measuring instrument and the measured parts are shown in Figure 10.

### 5.2. Examples of K-GDES Application

The K-GDES is experimentally validated using a batch of grinding surfaces and a batch of milling surfaces. The grinding surfaces are generally smooth but combined with obvious local random error, which may be due to the measurement process. The data of the milling surfaces are even rougher, with a large number of high-frequency waves covering the machining features. There are significant differences between two types of machining surfaces, so these two batches of surfaces can be used to demonstrate the applicability of K-GDES under different conditions. The software used for data processing and visualization in this paper is Matlab R2022b.

#### 5.2.1. Verification Based on Grinding Surfaces

**Figure 11:**
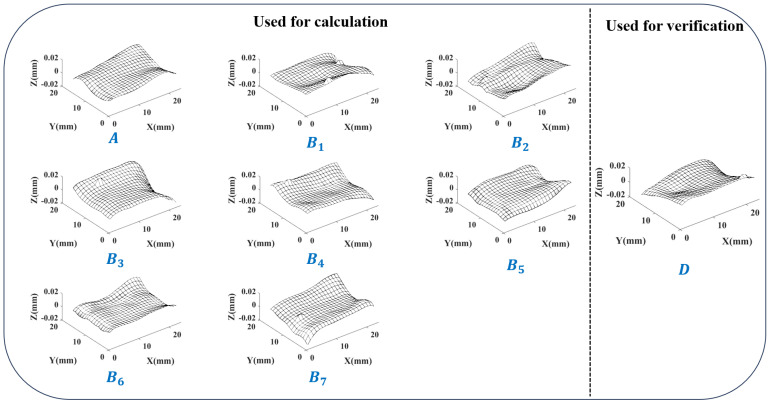
A batch of grinding surfaces after preprocessing.

Firstly, taking a batch of 9 grinding surfaces obtained using the same grinding machine and machining parameters as an example. Number them as $A,B_{1},\ldots ,B_{7},D$. The measurement data of the grinding surfaces are overall smooth but with a few local random errors. The error surfaces obtained after preprocessing are shown in Figure 11. Among them, 8 surfaces are used to calculate the error distribution similarity, including selected surface *A* and 7 comparison surfaces $B_{1},\ldots ,B_{7}$. The last surface *D* is not involved in the calculation of error distribution similarity but is subsequently separated in the same way to act as additional validity verification.

**Figure 12:**
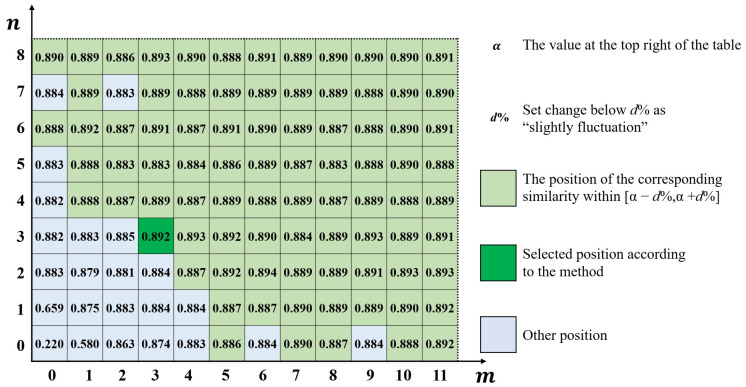
Error distribution similarity table for a batch of grinding surfaces.

**Figure 13:**
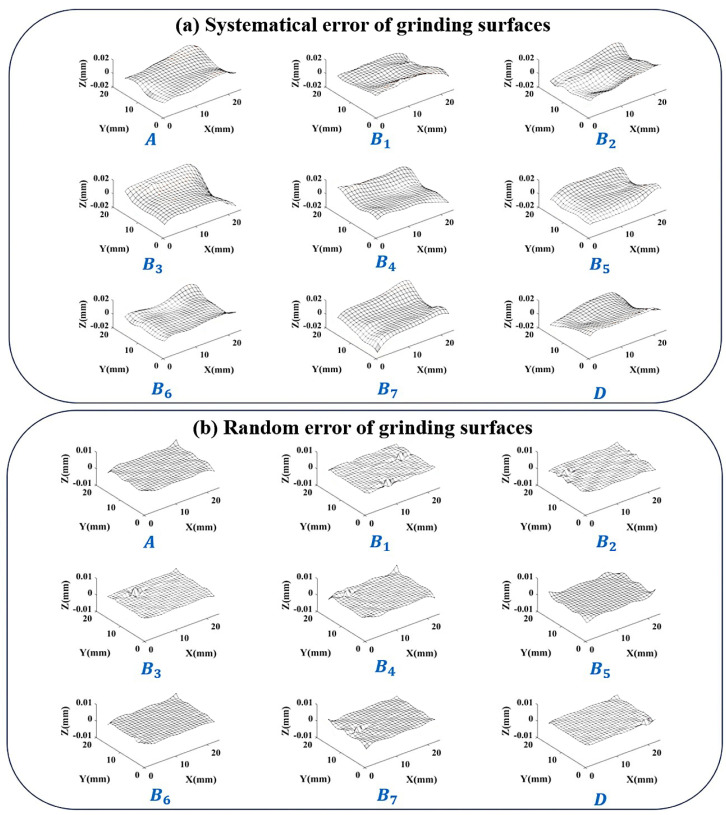
Separation results of a batch of grinding surfaces.

Using the method in Section 4.2.3, the calculated error distribution similarity table is shown in Figure 12:

Taking 0.5% as a threshold, there is α=89.1% in the upper right corner, so choose the interval [88.6%,89.6%]. Consider the number of elements contained within the boundary line, and finally, select the boundary position (3,3). After separating according to this position, 9 sets of results are obtained, as shown in Figure 13.

The significant local changes in the error surface are successfully included in random error. Analyze some indicators of the separation results:

1. The error distribution similarity between the systematical error surface and the original error surface $\eta_{0}$
  Shows the similarity between the surface composed of systematical error and the surface containing all error. The average value of $\eta_{0}$ between the 8 error surfaces involved in the calculation and their respective systematical error surfaces is 96.32%. And the $\eta_{0}$ of validation surface *D* is 97.02%. A high degree of similarity indicates that the error component of the control surface shape has been successfully segmented into systematical error parts.
2. The proportion of systematical error amplitude to the total error amplitude ε
  This indicator represents the proportional relationship between the separated systematical error and random error. Calculate the proportion of systematical error in the K-space amplitude spectrum of the error surface to the total error amplitude. The average ε of the 8 error surfaces participating in the calculation is 88.45%. And the ε of validation surface *D* is 93.22%. In this group of grinding surfaces, due to the low random error components, the ε value is relatively high.
3. Random error peak to peak value PP
  The D-value between the highest and lowest points of the Z coordinate in the separated random error. The average value PP of the 8 error surfaces in the calculation is 4.6 μm, and the PP of validation surface *D* is 2.9 μm. The magnitude of random error is in the micrometer range.

Based on the separation results and indicators, it is found that the systematical error surface obtained under K-GDES has little change in shape compared to the original error surface. The division of random error part is reasonable and can properly handle situations where the overall error surface is smooth but with obvious local random error.

#### 5.2.2. Verification Based on Milling Surfaces

**Figure 14:**
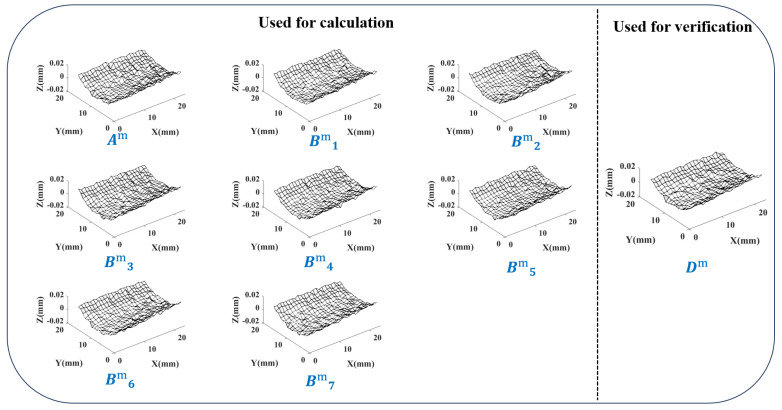
A batch of milling surfaces after preprocessing.

Then, also taking a batch of 9 milling surfaces obtained using the same milling machine and machining parameters as an example. Number them as $A^{m},{B^{m}}_{1},\ldots ,{B^{m}}_{7},D^{m}$. The precision of milling surfaces is slightly lower, and the random error amplitude is larger and more widely distributed than grinding surfaces. If the error separation is not reasonable, it is very easy to cover up the information of circular tool marks. This section tests the retention ability of K-GDES for circular or strip machining features in machining. The error surfaces obtained after preprocessing are shown in Figure 14. Among them, 8 surfaces are used to calculate the error distribution similarity, including selected surface $A^{m}$ and 7 comparison surfaces ${B^{m}}_{1},\ldots ,{B^{m}}_{7}$. The last surface $D^{m}$ does not participate in the calculation of error distribution similarity but is subsequently separated in the same way to act as additional validity verification.

**Figure 15:**
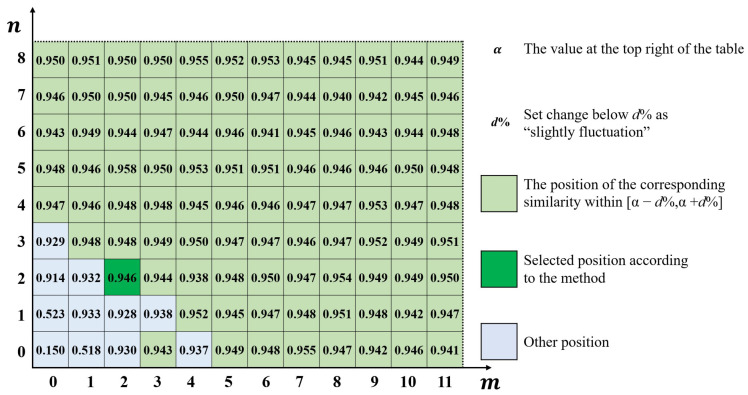
Error distribution similarity table for a batch of milling surfaces.

Using the method in Section 4.2.3, the calculated error distribution similarity table is shown in Figure 15.

**Figure 16:**
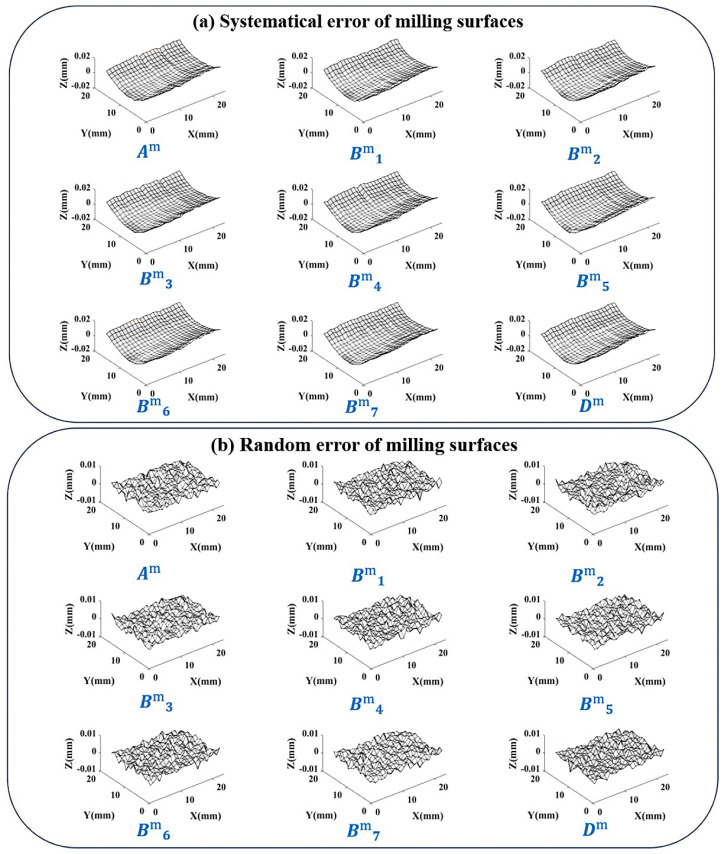
Separation results of a batch of milling surfaces.

Due to the large amplitude of random error in this batch of milling surfaces, the error distribution similarity fluctuates violently. So taking 1% as threshold, there is α=94.9% in the upper right corner, so choose the interval [93.9%,95.9%]. Consider the number of elements contained within the boundary line, and finally select the boundary position (2,2). After separating according to this position, 9 sets of results are obtained, as shown in Figure 16.

It can be seen that the systematical error not only retains the main trend of central depression, but also successfully preserves the information of circular tool marks caused by the milling cutter. Analyze some indicators of the separation results:

1. The error distribution similarity between the systematical error surface and the original error surface $\eta_{0}$
  The average value of $\eta_{0}$ between the 8 error surfaces involved in the calculation and their respective systematical error surfaces is 94.54%. And the $\eta_{0}$ of validation surface $D^{m}$ is 92.63%. A high degree of similarity indicates that the error component of the control surface shape has been successfully segmented into systematical error parts.
2. The proportion of systematical error amplitude to the total error amplitude ε
  The average ε of the 8 error surfaces participating in the calculation is 69.52%. And the ε of validation surface $D^{m}$ is 59.57%. Compared to grinding surfaces, the random error amount in milling surfaces significantly increases, so the proportion of systematical error decreases, which is consistent with the actual situation.
3. Random error peak to peak value PP
  The average value PP of the 8 error surfaces in the calculation is 7.8 μm, and the PP of validation surface $D^{m}$ is 7.2 μm. The random error peak-to-peak values of milling surfaces are higher than those of grinding surfaces because the milling accuracy is lower than that of grinding, which is consistent with the actual situation.

It is found that when the amount of random error is large, the similarity between the obtained systematical error surface and the original error surface slightly decreases. However, the K-GDES method still achieves smoothing while preserving as many strip-shaped and circular tool marks as possible.

### 5.3. Stability Analysis of K-GDES

**Figure 17:**
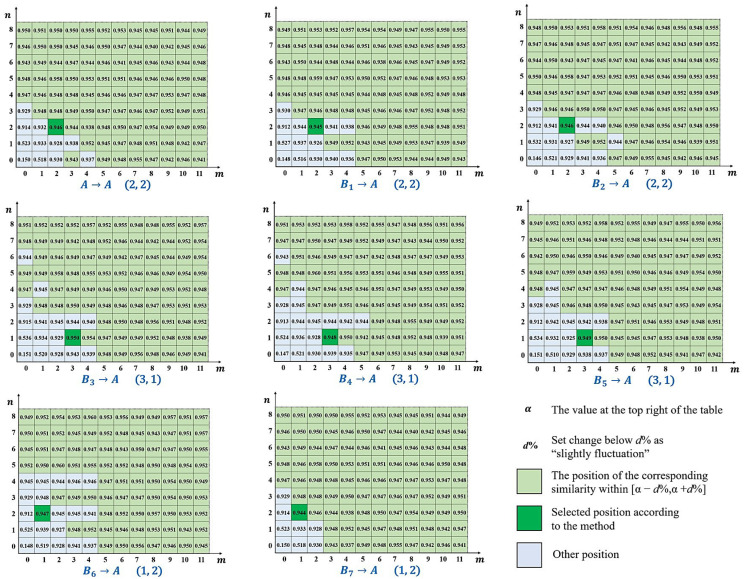
Error distribution similarity tables obtained from transforming *A*.

In this method, there are human factors that affect the results of error separation, specifically the selection of selected surface *A* in Section 4.2.3, and this impact is analyzed as follows. Currently, the selected surface *A* in this method is randomly selected, and replacing other surfaces as selected surfaces will result in different error distribution similarity tables, which will affect the optimal boundary position selected. Based on the example of grinding surfaces in Section 5.2.1, the 8 error surfaces participating in the calculation of error distribution similarity are sequentially used as selected surface, and the 8 error distribution similarity tables are obtained. The error distribution similarity table obtained after transforming the selected surface is shown in Figure 17, where $B_{k}\to A$ represents changing the comparison surface $B_{k}$ to the selected surface *A* and adding the original selected surface to the comparison surfaces.

Adopting a threshold of 0.5%, in addition to the previous boundary positions (3,3), there are also boundary positions (4,3). The comparison of various indicators after separating the validation surface *D* at each boundary position is shown in Table 1.

Out of the 8 selections, there are 6 occurrences (3,3) and 2 occurrences (4,3). And the difference in results after separation is not significant.

In summary, the selection of the selected surface *A* will have an impact on the results of this method, but the degree of this impact is limited. If we want to minimize its impact, a useful way is to take the average of the 8 error distribution similarity tables and then select the optimal boundary position.

## 6. Discussion

To analyze the advantages and disadvantages of K-GDES, a comparison is made with commonly used filtering methods such as low-pass filtering, wavelet filtering, Gaussian filtering, Wiener adaptive filtering, bilateral filtering, and other methods.

**Figure 18:**
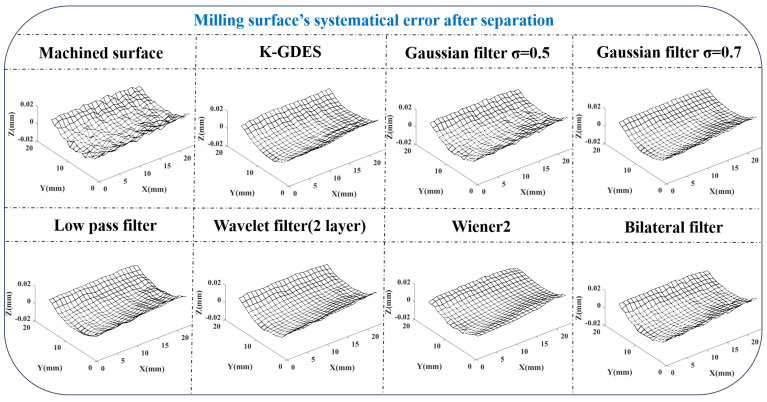
Comparison of the effectiveness of K-GDES with other methods.

Due to the smoothness of grinding surfaces, the separation results lack significant differences under these different methods. In this case, the milling surfaces are used as the comparison objects for these methods. Due to the lack of standard answers for separation results of GDE, it is not possible to calculate standard filtering indicators such as SSIM. Therefore, the random error peak-to-peak value PP is used to indicate the filtering strength and the error distribution similarity between the systematical error surface and the original error surface $\eta_{0}$ is used to indicate the degree of change in the shape behind the filter. The effects of different methods are shown in Figure 18.

In terms of indicator data, there is a Table 2.

Analyze the results of each method. The K-GDES method is similar in filtering strength to Gaussian filtering and low-pass filtering at σ = 0.7. However, the Gaussian filter at σ = 0.7 exhibits excessive smoothing, while the low-pass filter exhibits an unexpected ripple shape. Gaussian filtering at σ = 0.5 results in incomplete removal of random error. The two-layer wavelet filter has the smallest degree of surface shape change and performs well in data smoothing but eliminates circular tool marks. Wiener adaptive filtering does not require adjusting parameters, but it is evident that its results exhibit excessive smoothing, meaning that the adaptive method is severely ineffective in GDE processing. The bilateral filtering method essentially performs approximately Gaussian filtering in this case, with no significant difference in results.

Gaussian filtering, low-pass filtering, wavelet filtering, and bilateral filtering all require parameter adjusting. In the Gaussian filtering method, changing σ = 0.5 to σ = 0.7 results in a significant difference in separation results. The wavelet basis and filtering function in wavelet filtering need to be adjusted. The parameter adjustment of bilateral filtering is further complicated based on Gaussian filtering. K-GDES and Wiener adaptive filtering do not require parameter adjustment, but Wiener adaptive methods perform poorly in GDE processing.

It can be observed that at low data densities (such as the 1 mm measurement data interval in this example), K-GDES has a better ability to preserve machining features, especially strip-like features. Due to the tendency of milling and cutting to leave strip-shaped features, K-GDES is more suitable for error handling of precision machining surfaces than other methods. However, if the grinding surface in Section 5.2.1 is used as an example to compare the effects of various methods, we can find that when the amplitude of random error is significantly smaller than the processing features, the difference in effect between K-GDES and smoothing methods such as Gaussian filtering is not significant.

In conclusion, when processing GDE for key surfaces of components, according to experimental verification and comparison, the advantages of K-GDES are as follows:

1. Unified error separation of a batch of machining surfaces is achieved under the same processing method;
2. Can still be used normally under low data density;
3. When there is a certain periodic pattern in the machining features, the ability to preserve the machining features is excellent;
4. Due to the fact that the method is based on frequency, it can still be used normally when the amplitude of high-frequency random error is high.

At the same time, this method has limitations:

1. The effectiveness of K-GDES under high data density has not been verified and compared with other methods;
2. A batch of machining surfaces is required, so it is not applicable to a single surface;
3. When the magnitude of the random error is much lower than systematical error, K-GDES does not have a significant advantage over smoothing methods such as the Gaussian filter method;
4. Difficult to handle low-frequency random errors that may exist in measurement data.

## 7. Conclusions

After preprocessing, frequency-domain representation, identification, and separation, the K-GDES method proposed in this article can achieve accurate systematical error and random error separation for a batch of machined surfaces. By utilizing the K-space spectrum, error separation suitable for machining surfaces can be achieved at low data densities. By analyzing and quantifying systematical error and random error, a quantitative evaluation of the machining accuracy of the workpiece can be achieved. By separating and highlighting machining features, more suitable models for simulation and analysis can be constructed.

The K-GDES method can still be further improved in some cases. If a much higher density of high-precision measurement data can be obtained, we will be able to further distinguish the shape error, waviness, and roughness. In this case, the idea of avoiding parameter adjustment by analyzing the similarity relationship between error surfaces in the same batch is worth preserving and extending. It can incorporate the idea of wavelet filtering and deal with more complex data situations by performing more refined processing in the frequency domain. In addition, current construction methods for models essentially involve constructing individual models for a batch of components. More than one result is obtained. If a single representative model can replace this batch of models, it will be more conducive to the application. This article currently does not cover how to transition from multiple results to a single representative model, but we will further investigate specific methods in the future.

## Figures and Tables

**Table 1:** Comparison of separation result of different separation boundary positions.

(m,n)	(3,3)	(4,3)
$\eta_{0}$	97.02%	96.47%
ε	93.22%	94.50%
PP	2.9 μm	2.5 μm

**Table 2:** Comparison of indicators between K-GDES and other methods.

Method Category	$\eta_{0}$	PP
K-GDES	93.69%	7.1 μm
Gaussian σ = 0.5	94.39%	3.3 μm
Gaussian σ = 0.7	92.63%	7.2 μm
Low pass filter	93.02%	7.9 μm
Wavelet filter	96.06%	8.3 μm
Wiener2	91.83%	11.1 μm
Bilateral filter	94.23%	4.0 μm

## Data Availability

This article uses some measurement data of machining surfaces, which have not been uploaded yet. The raw data supporting the conclusions of this article will be made available by the authors on request.

## Abbreviations

The following abbreviations are used in this manuscript:

GDE	Geometric Eistribution Error
K-GDES	An error separation method for GDE based on K-space spectrum
